# Epidemiological, clinical, and microbiological characteristics of carbapenemase-producing Enterobacteriaceae bloodstream infection in the Republic of Korea

**DOI:** 10.1186/s13756-019-0497-3

**Published:** 2019-03-05

**Authors:** Jung-wan Park, Hyungmin Lee, Se Yoon Park, Tae Hyong Kim

**Affiliations:** 1 Department of Infectious Disease, Asan Medical Center, University of Ulsan College of Medicine, 88, Olympic-ro 43-gil, Songpa-gu, Seoul, 05505 Republic of Korea; 2 Department of Healthcare Associated Infection Control, Korea Centers for Disease Control and Prevention, 187, Osongsaengmyeong 2-ro, Osong-eup, Heungdeok-gu, Cheongju-si, Chungcheongbuk-do 28159 Republic of Korea; 3 Division of Infectious Diseases, Department of Internal Medicine, Soonchunhyang University Seoul Hospital, Soonchunhyang University College of Medicine, 59, Daesagwan-ro, Yongsan-gu, Seoul, 04401 Republic of Korea

**Keywords:** Infection, Carbapenemase-producing Enterobacteriaceae, Republic of Korea, *Klebsiella*, Nosocomial infection

## Abstract

**Background:**

Carbapenemase-producing Enterobacteriaceae (CPE) is an important pathogen in nosocomial infections; nevertheless, only a few studies regarding CPE infection and its epidemiological factors have been conducted in the Republic of Korea (ROK). We aimed to analyze the clinical, microbiological, and epidemiological characteristics of CPE bloodstream infections (BSIs) in the ROK.

**Methods:**

This retrospective cohort study included data collected from the National Surveillance System from January 2015 to December 2016 based on the epidemiologic survey performed by an epidemiologist from the Korea Centers for Disease Control and Prevention. We selected patients with CPE BSI from the Korea National Institute of Health based on carbapenemase genotyping.

**Results:**

In this study, 131 CPE BSIs were identified, and the proportion of CPE BSI among total CPE isolates was 7%. *Klebsiella pneumoniae* accounted for 69% of all CPE BSIs, and 66% of these produced *K. pneumoniae* carbapenemase. Among nine provinces in ROK, one province had NDM as the most common carbapenemase. CPE was susceptible to amikacin, tigecycline, and gentamicin (76, 41, and 39%, respectively). Of 29 patients tested for colistin sensitivity, one patient showed colistin resistance. The most common CPE BSI sources were pneumonia, primary bacteremia, and biliary tract infection. Multivariable analysis showed that adequate antibiotic use at CPE detection was significantly associated with decreased 30-day mortality.

**Conclusions:**

CPE BSIs are prevalent in the ROK. Moreover, most CPE BSIs originated from hospital-acquired infection, demonstrating the need to improve hospital infection control strategies.

**Electronic supplementary material:**

The online version of this article (10.1186/s13756-019-0497-3) contains supplementary material, which is available to authorized users.

## Background

Enterobacteriaceae is one of the major contributors to nosocomial infections. *Klebsiella pneumoniae* and *Enterobacter* species infections account for 15–51% of all nosocomial infections in intensive care units [1, 2]. Furthermore, there has been a gradual increase in the risk for multidrug resistance among Enterobacteriaceae, including carbapenemase-producing Enterobacteriaceae (CPE) [3, 4].

Since the first case of CPE was reported in 2010 in the Republic of Korea (ROK) (*5*), the number of patients with CPE infection or those carrying CPE has increased exponentially. Several medical institutions have experienced CPE outbreaks due to transmission within healthcare settings [6]. With the rise in the number of CPE infection cases, the associated disease burden is also increasing [7]. The 30-day mortality of CPE bloodstream infection (BSI) is approximately 50%, despite the application of empirical treatment [8]. The mortality associated with CPE infection is four times higher than that associated with non-carbapenemase-producing carbapenem-resistant Enterobacteriaceae (non-CP-CRE) [9].

Due to the high rate of proliferation and virulence associated with CPE, the importance of preventing CPE transmission has been emphasized [10, 11]. The Korea Centers for Disease Control and Prevention (KCDC) has been conducting surveillance of CPE infection/carrier cases. The KCDC expanded the range of surveillance in June 2017, before which sentinel surveillance was performed. However, data on CPE infection in the ROK were limited.

Herein, we aimed to describe the epidemiological, clinical, and microbiological characteristics of CPE BSIs in the ROK. We also aimed to investigate the risk factors for mortality associated with CPE BSIs.

## Materials and methods

### Study setting and data collection

All CPE isolates obtained from patients at sentinel medical institutions (100 institutions in 2015, and 115 in 2016) were mandatorily reported to the KCDC through the National Surveillance System. An epidemic investigation officer from the KCDC produced epidemic investigation reports on CPE infection/carrier cases by reviewing patients’ medical records including data on clinical features, microbiological data, epidemiological characteristics, and outcomes. We retrospectively reviewed the CPE BSI cases using data collected through the National surveillance system from January 2015 to December 2016, based on the epidemiologic survey performed by the epidemiologist from the KCDC. In particular, we investigated the antibiotic susceptibility of CPE BSI.

### Confirmation of CPE

All sentinel laboratories used automated systems for microorganism identification and in vitro antibiotic susceptibility testing. When sentinel laboratories reported CRE, they also sent samples of specimens containing CPE to National Institute Health (NIH) in Korea, and the NIH confirmed the presence of CRE. When a sentinel medical institution detected CRE, the isolated specimen was sent to the NIH for confirmatory testing. The NIH performs antibiotic susceptibility tests according to the Clinical and Laboratory Standards Institute guidelines, while resistance is judged based on M100-S27 (2017) [12]. They followed the CDC’s “Laboratory Protocol for Detection of Carbapenem-Resistant or Carbapenemase-Producing, *Klebsiella* spp. and *Escherichia coli* from Rectal Swabs” [13]. The examination was performed based on the following procedure: all acquired specimens were cultivated in Trypticase soy broth mixed with carbapenem. The cultured fluid was streaked on MacConkey agar and cultivated once more. Then, the isolated colony was determined by phenotype test like mixed hemagglutination test or carbapenem minimum inhibitory concentration (MIC). If carbapenem resistance was confirmed, a carbapenemase gene polymerase chain reaction (PCR) or sequencing was performed to detect the presence of carbapenemase. A modified Hodge test was performed in all the commissioned specimens, and if the result was positive, a genotyping test was performed to confirm the types of carbapenemase.

### Definition

We defined CPE as CRE with any carbapenemase (KPC, NDM, IMP, GES, VIM, or OXA-48), while CRE was defined based on the KCDC guidelines for CPE control, as confirmed by the MIC of each carbapenem: the MIC of doripenem, imipenem, and meropenem was ≥4 μg/ml, while the MIC of ertapenem was ≥2 μg/ml [12].

Hospital-acquired infections were confirmed through the positive blood culture results of patients who were hospitalized for more than 48 h. Community-acquired or healthcare-associated infections were confirmed among patients who had BSI from blood culture samples within 48 h. If they had a history of hospitalization history in the last 90 days or were transferred from another medical institution, the infection was defined as healthcare associated. If the patient developed BSI within 48 h without history of hospitalization in the last 90 days, community-acquired infection was confirmed.

Empirical therapy was defined as the antimicrobial therapy administered before the bacterial strain was identified and before the susceptibility report was provided by the hospital laboratory [14]. We defined the adequate treatment for CPE infection if the physician used susceptible antibiotics in isolated bacteria in vitro or if the physician chose a combination therapy, at least one drug was active in vitro against the infecting organism [15]. If a clinician used an antibiotic to treat CPE BSI, in which the susceptibility was confirmed by an antibiogram, the antibiotic therapy was judged as being adequate.

Patients who died within 30 days from CPE BSI occurrence were defined as “non-survivors.” The clinical data of the patients were collected at the time of CPE BSI detection.

Extended-spectrum beta-lactamase was detected by performing an extended-spectrum beta-lactamase test with disk diffusion or broth microdilution using an automated susceptibility system according to the guidelines of the Clinical and Laboratory Standards Institute [12]. We confirmed ESBLs using automated equipment in each hospital.

### Calculation of the proportion of CPE BSI among total CPE isolates

To calculate the proportion of CPE BSI among CPE isolates, we used the data on total CPE isolates from the Public Health Weekly report, *“Status of carbapenemase-producing enterobacteriaceae incidences in Korea, 2015–2016”* [6]. This report contains data on the total number of CPE infection/carrier cases as obtained through national sentinel surveillance, regional occurrences, and microbiological features.

### Statistical analysis

SPSS version 21.0 for Windows (IBM Corporation, Armonk, NY) was used to perform all statistical analyses. Categorical variables were analyzed by a Chi-square test or Fisher’s extract test, as appropriate. Continuous variables were analyzed by an independent samples t-test or a Mann-Whitney U test. Logistic regression analysis was performed to evaluate the effect of independent variables on risk. A two-tailed *P*-value of < 0.05 was considered significant. All variables with *P* values lower than 0.05 in the univariate analysis were included in the multiple logistic regression models in which we identified the risk factors for mortality due to CPE BSIs.

## Results

### Epidemiologic characteristics of CPE BSIs in the ROK

A total of 2020 CPE isolates were obtained from January 2015 to December 2016. There were 131 cases of CPE BSI (proportion: 6.5%). Approximately 67% of the CPE BSI cases were reported in advanced general hospitals. The regional distributions and differences between the microbiological features are described in Table 1. About 48.9% of the CPE BSI patients were from Seoul; 26.7% were from Busan, Ulsan, and Gyeongsangnam-do; and 19.8% were from Incheon and Gyeonggi-do. The proportion of CPE BSI among total CPE isolates were not significantly different between each region and nationwide (*P* = 0.08 (Seoul), 0.41 (Busan, Ulsan, and Gyeongsangnam-do), 0.21 (Gyeongangbuk-do and Daegu), 0.63 (Chungcheong-do and Daejeon), 0.21 (Jeolla-do and Gwangju), and 0.64 (Gangwon-do)) except for Incheon and Gyeonggi-do (*P* = 0.01).

**Table 1:** Epidemic characteristics of carbapenemase-producing Enterobacteriaceae bloodstream infection

	Total	A	B	C	D	E	F	G
CPE bloodstream infection	131	64 (49)	35 (27)	26 (20)	2 (2)	2 (2)	1 (1)	1 (1)
Hospital type								
General hospital	43 (33)	8 (13)	24 (69)	8 (31)	1 (50)	1 (50)	0	1 (100)
Advanced general hospital	88 (67)	56 (88)	11 (31)	18 (69)	1 (50)	1 (50)	1 (100)	0
*Enterobacteriaceae* type								
*Escherichia coli*	10 (8)	6 (9)	2 (6)	1 (4)	0	0	1 (100)	0
*Klebsiella pneumoniae*	90 (69)	42 (66)	25 (71)	21 (81)	0	1 (50)	0	1 (100)
*Enterobacter* species	13 (10)	7 (11)	2 (6)	2 (8)	2 (100)	0	0	0
*Klebsiella oxytoca*	7 (5)	7 (11)	0	0	0	0	0	0
*Citrobacter* species	4 (3)	1 (2)	1 (3)	2 (8)	0	0	0	0
*Serratia marcescens*	7 (5)	1 (2)	5 (14)	0	0	1 (50)	0	0
Carbapenemase type								
OXA-48	7 (5)	3 (5)	0	2 (8)	0	0	1 (100)	1 (100)
NDM	26 (20)	13 (20)	1 (3)	12 (46)	0	0	0	0
KPC	86 (66)	40 (63)	34 (97)	11 (42)	0	1 (50)	0	0
VIM	8 (6)	7 (11)	0	0	1 (50)	0	0	0
IMP	2 (2)	1 (2)	0	1 (4)	0	0	0	0

Abbreviations: *OXA* Oxacillin carbapenemases, *NDM* New Delhi metallo-beta-lactamase enzyme**,** *KPC* Klebsiella pneumoniae Carbapenemase, *VIM* Verona integron-encoded metallo-beta-lactamase, *IMP* Imipenemase metallo-beta-lactamase, *CPE* Carbapenemase-producing *Enterobacteriaceae*

Note: Data presented are numbers (%) of patients, unless otherwise indicated

A: Seoul, B: Busan, Ulsan, and Gyeongsangnam-do, C: Incheon and Gyeonggi-do, D: Gyeongangbuk-do and Daegu, E: Chungcheong-do and Daejeon, F: Jeolla-do and Gwangju, G: Gangwon-do

Nationwide, the proportion of *K. pneumoniae* BSI was the highest (69%), followed by that of *Enterobacter* spp*.* and *E. coli* infections (10 and 8%, respectively). In the regional analysis, the Enterobacteriaceae types were similar to those observed nationwide. Specifically, *Serratia marcescens* was prevalent in Busan, Ulsan, and Gyeongsangnam-do (14%), while *Citrobacter* spp. was prevalent in Incheon and Gyeonggi-do (8%). With regard to the types of carbapenemase, KPC was the most commonly observed in the ROK (66%) followed by NDM (20%). The patterns of carbapenemase distribution in Seoul were similar to those observed nationwide; however, in Busan, Ulsan, and Gyeongsangnam-do, KPC was extremely dominant compared with the other types of carbapenemase. In Incheon and Gyeonggi-do, NDM was more dominant than KPC (46 and 42%, respectively).

Data on infection acquisition are described in Table 2. A total of 111 (85%) patients had hospital-acquired CPE BSI. About 48 (43%) of the infections occurred during CPE outbreak (data not shown). A total of 15 (12%) patients had healthcare-associated infections, and 5 (33%) were transferred from nursing hospitals. About 5 (4%) patients were suspected of having community-acquired infection. The mode of transmission did not affect mortality (*P* = 0.08).

**Table 2:** Clinical characteristics of carbapenemase-producing Enterobacteriaceae bloodstream infection and antibiotics applied for its treatment

Characteristics	Total (*N*=131)	Survivor (*N*=69)	Non-survivor (*N*=62)	P
Age ± SD (years)	59.6±18.9	57.8±18.5	61.6±19.2	0.23
Male	86 (66)	46 (67)	40 (65)	0.25
Transfer history	47 (36)	24 (35)	23 (37)	0.93
Acquisition of infection				0.08
Community	5 (4)	5 (7)	0	0.06
Healthcare-associated	15 (12)	8 (12)	7 (11)	0.96
Hospital-acquired	111 (85)	56 (81)	55 (89)	0.23
Comorbidity				
Total	112 (86)	62 (90)	50 (81)	0.21
DM	37 (28)	16 (23)	21 (34)	0.25
Solid organ cancer	34 (26)	18 (26)	16 (26)	1.00
Hematology cancer	33 (25)	20 (29)	13 (21)	0.39
ESRD	21 (16)	11 (16)	10 (16)	1.00
Heart disease	15 (12)	7 (10)	8 (13)	0.83
Liver disease	13 (9)	7 (10)	6 (10)	1.00
Stroke	12 (9)	7 (10)	5 (8)	0.91
Immunosuppressive therapy state	6 (5)	2 (3)	4 (7)	0.58
COPD	2 (2)	0	2 (3)	0.43
Charlson comorbidity index	4.7±3.0	4.4±2.8	5.0±3.3	0.33
Portal of entry				
Pneumonia	38 (29)	14 (20)	24 (39)	0.02
Primary bacteremia	21 (16)	12 (17)	9 (15)	0.65
Cholangitis/cholecystitis	20 (15)	11 (16)	9 (15)	0.82
Skin and soft tissue infection (include post operation site infection)	14 (11)	7 (10.1)	7 (11.3)	0.83
UTI	13 (10)	11 (16)	2 (3)	0.02
Unknown	10 (8)	5 (7)	5 (8)	0.86
Intraabdomen infection	8 (6)	5 (7)	3 (5)	0.72
catheter-related BSI	7 (5)	4 (6)	3 (5)	1.00
Operation				
General	41 (31)	17 (25)	24 (39)	0.12
Minor	29 (22)	13 (19)	16 (26)	0.45
Simple	84 (64)	41 (59)	43 (69)	0.32
Tracheostomy	25 (19)	11 (16)	14 (23)	0.46
Intensive care unit admission	74 (57)	29 (42)	45 (73)	0.001
Invasive catheter	124 (95)	63 (91)	61 (98)	0.16
Mechanical ventilation	60 (46)	22 (32)	38 (61)	0.001
APACHE II score	17.2±8.0	12.8±5.4	22.2±7.6	<0.001
Treatment				
Adequate antibiotic therapy	106 (82)	62 (90)	46 (74)	0.03
Amikacin-based therapy	33 (31)	17 (27)	16 (38)	0.53
Colistin-based therapy	58 (54)	28 (45)	30 (65)	0.05

Note: Data presented are numbers (%) of patients, unless otherwise indicated

Abbreviation: *SD* Standard deviation, *DM* Diabetes mellitus, *COPD* Chronic obstructive pulmonary disease, *UTI* Urinary tract infection, *APACHE* Acute Physiology and Chronic Health Evaluation, *CPE* Carbapenemase-producing *Enterobacteriaceae*, *TMP/SMX* Trimethoprim/sulfamethoxazole

### CPE antibiotic susceptibility

The antibiotics susceptible to CPE are shown in Table 3 and Additional file 1 Table S1. A total of 99 cases were sensitive to amikacin (75.6%), 54 were sensitive to tigecycline (41.2%), and 51 were sensitive to gentamicin (38.9%). Approximately 47% of all CPE patients did not have extended-spectrum beta-lactamase (data not shown). Twenty-nine patients from nine medical institutions underwent colistin sensitivity testing, and one patient who was transferred from Dubai, United Arab Emirates, developed colistin resistance.

**Table 3:** Differences in antibiotic susceptibility by Enterobacteriaceae type

Antibiotics susceptibility	Total (*N*=131)			*Escherichia coli* (*N*=10)	*Klebsiella pneumoniae* (*N*=90)	*Enterobacter* spp. (N=13)	*Klebsiella oxytoca* (*N*=7)	*Citrobacter* spp. (*N*=4)	*Serratia marcescens* (*N*=7)
	Susceptible	Intermediate	Resistant						
Ampicillin	1 (1)	0	130 (99)	0	1 (1)	0	0	0	0
Ampicillin/sulbactam	0	0	131 (100)	0	0	0	0	0	0
Amikacin	99 (76)	2 (2)	30 (23)	9 (90)	66 (73)	9 (69)	6 (86)	4 (100)	5 (71)
Aztreonam	6 (5)	0	125 (95)	3 (30)	0	0	3 (43)	0	0
Ceftazidime	3 (2)	0	128 (98)	0	0	0	1 (14)	0	2 (29)
Cefotaxime	1 (1)	0	130 (99)	1 (10)	0	0	0	0	0
Ciprofloxacin	18 (14)	3 (2)	110 (84)	1 (10)	7 (8)	3 (23)	4 (57)	2 (50)	1 (14)
Cefuroxime	0	0	131 (100)	0	0	0	0	0	0
Cefazoline	0	0	131 (100)	0	0	0	0	0	0
Cefepime	8 (6)	2 (2)	121 (92)	0	2 (2)	2 (15)	3 (43)	0	1 (14)
Cefoxitin	1 (1)	0	130 (99)	0	1 (1)	0	0	0	0
Gentamicin	51 (39)	12 (9)	68 (52)	5 (50)	29 (32)	5 (39)	2 (29)	3 (75)	7 (100)
Levofloxacin	12 (9)	4 (3)	115 (88)	1 (10)	3 (3)	4 (31)	3 (43)	0	1 (14)
Tobramycin	4 (3)	4 (3)	123 (94)	1 (10)	1 (1)	2 (15)	0	0	0
Piperacillin/tazobactam	1 (1)	5 (4)	125 (95)	0	0	0	0	0	1 (14)
TMP/SMX	30 (23)	0	101 (77)	2 (20)	18 (20)	1 (8)	2 (29)	1 (25)	6 (86)
Tigecycline	54 (41)	9 (7)	68 (52)	7 (70)	37 (41)	2 (15)	4 (57)	2 (50)	2 (29)
Tetracycline	12 (9)	1 (1)	118 (90)	1 (10)	9 (10)	1 (8)	1 (14)	0	0
Ticarcillin/clavulanate	1 (1)	1 (1)	129 (99)	0	1 (1)	0	0	0	0
Colistin*	28/29 (97)	0/29	1/29 (3)	4/4 (100)	18/19 (95)	1/1 (100)	3/3 (100)	2/2 (100)	0/0

Note: Data are numbers (%) of patients, unless otherwise indicated

Abbreviation: spp. - Species, TMP/SMX - Trimethoprim/sulfamethoxazole

* Only 29 cases were performed colistin sensitivity test

We demonstrated the antibiotic susceptibility patterns according to the type of Enterobacteriaceae. *K. oxytoca* was more sensitive to aztreonam, ciprofloxacin/levofloxacin, and cefepime than *K. pneumoniae* (*P* < 0.001, 0.001, < 0.001, and < 0.001, respectively). *S. marcescens* was more sensitive to trimethoprim/sulfamethoxazole (86%) than other organisms (*P* < 0.001).

When we analyzed the antibiotic susceptibility of KPC-producing CPE or MBL-producing CPE, there was no significant difference in aztreonam susceptibility between KPC and NDM (1.2% [1/86] in KPC-producing CPE and 3.8% [1/26] in NDM-producing CPE, *P* = 0.41) (Additional file 1 Table S2).

### Baseline characteristics of the survivors and non-survivors

Table 2 shows the patients’ baseline characteristics. The 30-day mortality associated with CPE BSI was 47%. The factors affecting 30-day mortality were presence of lung infection, presence of urinary tract infection, a low Acute Physiology and Chronic Health Evaluation (APACHE) II score, and history of antibiotic therapy for CPE infection (*P* = 0.02, 0.02, < 0.001, and 0.03, respectively).

In the multivariate analysis, the measurement of the APACHE II score at the time of CPE BSI (*P* < 0.001) and provision of adequate antibiotic therapy for CPE (*P* = 0.01) were significantly associated with 30-day mortality (Table 4).

**Table 4:** Multivariate analysis of the risk factors of mortality due to carbapenemase-producing Enterobacteriaceae bloodstream infection

Characteristics	Univariate analysis		Multivariate analysis	
	Unadjusted OR (95% CI)	P	Adjusted OR (95% CI)	P
Age	1.01 (0.99–1.03)	0.23	–	–
Pneumonia	2.48 (1.14–5.40)	0.02	–	–
UTI	0.18 (0.03–0.83)	0.02	–	–
Community-acquired infection	0.93 (0.87–0.99)	0.06	–	–
ICU admission	3.65 (1.75–7.61)	0.001	–	–
General operation	1.93 (0.91–4.09)	0.08	–	–
Mechanical ventilation	3.38 (1.65–6.95)	0.001	–	–
APACHE II score	1.25 (1.16–1.35)	< 0.001	1.23 (1.13–1.34)	< 0.001
Antibiotics therapy for CPE infection	0.27 (0.09–0.75)	0.03	0.12 (0.03–0.57)	0.007
Colistin based therapy	1.29 (0.65–2.58)	0.05	–	–
Combination therapy	0.54 (0.24–1.25)	0.15	–	–

Abbreviation: *OR* Odd ratio, *ICU* Intensive Care Unit, *UTI* Urinary tract infection, *APACHE* Acute Physiology and Chronic Health Evaluation, *CPE* Carbapenemase-producing *Enterobacteriaceae*, *No*. Number, *CI* Confidential index

The application of empirical antibiotics at the occurrence of the first sign of infection did not reduce mortality (*P* = 0.28). However, if physicians applied adequate antibiotics for the treatment of CPE BSI when they recognized that the pathogen had carbapenem resistance, the mortality significantly diminished (*P* = 0.03). The antibiotic regimens used for survivors and non-survivors were not significantly different. Moreover, combination antibiotic therapy did not decrease mortality (*P* = 0.68) (Additional file 1 Table S3).

## Discussion

To our knowledge, this study is the first to demonstrate the epidemiological, microbiological, and clinical features of CPE BSI patients in the ROK. Nationwide, the incidence of *K. pneumonia* with KPC-type carbapenemase was the highest, and most cases originated from hospital-acquired infections. CPE showed sensitivity to amikacin, tigecycline, and gentamicin. In this study, the 30-day mortality was 47%. A lower APACHE II score at CPE BSI occurrence or the application of adequate antibiotics for CPE was significantly associated with decreased mortality.

CRE is highly prevalent and is considered a public health threat worldwide [16]. According to an annual report of the Korean Antimicrobial Resistance Monitoring System, 2015, 1.7% of *K. pneumoniae*, 0.3% of *E. coli*, and 1.4% of *Enterobacter* species are carbapenem resistant, as observed in general hospitals nationwide [17]. Furthermore, the CPE infection incidence rate is increasing every year. According to the data from the KCDC, a total of 16 CPE infection cases were reported in 2011; however, 1455 CPE infection cases were observed in 2016 [6]. Around 40 medical institutions had experienced CPE outbreaks and more than two patients with CPE infection/CPE carriers with epidemiological relations were identified.

We found some regional differences in the data on CPE BSI occurrence. This could be attributed to the sentinel medical institutions’ characteristics. The number of medical institutions was the greatest in Seoul; moreover, the medical institutions in Seoul were relatively larger than those in the other regions. Therefore, CPE patients were concentrated in Seoul. Some medical institutions experienced large-scale CPE outbreaks in Seoul, Daegu, Busan, and Incheon; these events reflect our total data, including the microbiological features. In particular, the type of CPE, which was popular in most parts of Korea, was a KPC-producing CPE; however, in Incheon, most of the patients had the NDM type. This could be attributed to outbreaks from some medical institutions, and the fact that some CPE types were transmitted between hospitals.

Around 95% (126/133) of the patients in this study had hospital-acquired or healthcare-associated infections. There have been warnings on CPE outbreaks in medical institutions [5, 18]. According to the data on the CRE Epicenter of the United States, the rate of hospital-acquired infections was 66%; however, 75% of the patients who died had hospital-acquired infection [8]. In this study, 85% of all CPE BSI patients had hospital-acquired infection; about 43% of these patients acquired the infection during an outbreak in the hospital. This is why the importance of infection control is emphasized in hospitals.

Understanding the antibiotic susceptibilities of CPE is important for treatment-related decision-making. In the present study, a relatively higher susceptibility with aminoglycosides was noted (amikacin: 76% and gentamicin: 39%, respectively), which was consistent with those reported in previous studies [19, 20]. This finding could be attributed to the differences in the characteristics of various carbapenemase types. Similar data were reported by Zubair et al. [21], who analyzed 41 patients in whom KPC-type CPE bacteremia was detected; they found that only 14.6% of all CPE patients were susceptible to gentamicin. Moreover, NDM types could produce 16S ribosomal RNA methyltransferase, which leads to resistance to all aminoglycosides [22]. In terms of comprehensive treatment options, amikacin combination therapy may be an alternative plan to treat CPE infection when there is lack of adequate treatment options; however, our data did not reveal the effectiveness of this method in improving survival. More advanced research on the efficacy of amikacin combination treatment for CPE BSI should be performed.

Previous studies described the risk factors for mortality due to CPE infection to predict treatment outcomes. Mario et al. stated that the occurrence of septic shock, inadequate initial antimicrobial treatment, and a high APACHE II score increased mortality in patients with CPE BSI, but the application of combination therapy for CPE infection could decrease mortality [23]. Our data also suggested that patients’ underlying conditions including their APACHE II scores could be associated with mortality; however, the difference in mortality between different antibiotic regimens was small. This could be because patients with higher disease severity may have received combination therapy, compared with patients with mild-to-moderate disease. Most monotherapy regimens include colistin, which many CPE patients are susceptible; this could affect the survival of CPE BSI patients.

The strength of our study is that it is the first and largest research in the ROK to obtain data regarding the epidemiology, microbiology, and clinical features, including antibiotic susceptibility, of CPE bacteremia cases. However, our study has some limitations. First, as we used the data from epidemic investigation reports, only limited clinical data were obtained, such as duration of antibiotic therapy or data on why clinicians chose a particular antibiotic. Second, our data were obtained from sentinel surveillance. We only included those hospitals capable of monitoring and managing infectious diseases. Therefore, our findings cannot be generalized to smaller-sized hospital or clinics. Third, although most CPE BSI infections were hospital-acquired or healthcare-associated infections (96%, 126/131), we could not determine the carriage rate of CPE in patients with CPE BSI upon admission. In a previous study conducted between May 2016 and February 2017, in similar periods with that of our study in ROK, the CRE carriage rate of patients who transferred from long-term care facilities was 1.4% (4/282) without CPE colonizer [24]. Moreover, about one-third (37%) of the total CPE BSI cases occurred in the outbreak setting (data not shown). This finding suggests that most patients with CPE BSI currently have low CPE carriage rate. However, due to the rapidly changing CPE epidemiology in ROK, it is important to apply efficient CPE screening strategy in inpatients according to each institution’s epidemiology [25]. Fourth, carbapenem-sensitive or non-susceptible CPE may have been missed since the focus is on testing for presence of carbapenemase genes on carbapenem-resistant isolates only. This may underestimate the burden of CPE BSI.

## Conclusions

We found that the proportion of CPE BSI per total CPE isolates was high, nationwide, and most of these infections were transmitted in hospitals. The establishment of appropriate infection control strategies for medical institutions is important. Moreover, the application of adequate antibiotics including amikacin, gentamicin, or tigecycline could decrease the mortality associated with CPE BSI. More advanced research should be conducted in this regard.

## Additional file

Additional file 1: **Table S1.** Antibiotics susceptibility test depending on carbapenemase types. **Table S2.** Comparison of antibiotics susceptibility test between KPC and NDM. **Table S3.** Antibiotics regimen for treating carbapenemase-producing Enterobacteriaceae bloodstream infection. (DOCX 30 kb)

## Abbreviations

BSI: Bloodstream infection
CPE: Carbapenemase-producing Enterobacteriaceae
KCDC: Korea Centers for Disease Control and Prevention
MIC: Minimum inhibitory concentration
NIH: National Institute Health
ROK: Republic of Korea
