# Supplementary material for: Epidemiological, clinical, and microbiological characteristics of carbapenemase-producing Enterobacteriaceae bloodstream infection in the Republic of Korea
Source: Antimicrob Resist Infect Control. 2019 Mar 5;8:48. doi: 10.1186/s13756-019-0497-3 (PMC6402157; doi:10.1186/s13756-019-0497-3)
Supplement: Supplementary file 1 — Table S1. Antibiotics susceptibility test depending on carbapenemase types. Table S2. Comparison of antibiotics susceptibility test between KPC and NDM. Table S3. Antibiotics regimen for treating carbapenemase-producing Enterobacteriaceae bloodstream infection. (DOCX 30 kb) [file 13756_2019_497_MOESM1_ESM.docx]

Additional file 1: Table S1. Antibiotics susceptibility test depending on carbapenemase types

| Susceptibility | OXA-48  (N=7) | NDM  (N=26) | KPC  (N=86) | VIM  (N=8) | IMP  (N=2) | GES  (N=2) | Total  (N=131) |
| --- | --- | --- | --- | --- | --- | --- | --- |
| Ampicillin | 0 | 0 | 1 (1) | 0 | 0 | 0 | 1 (1) |
| Ampicillin/sulbactam | 0 | 0 | 0 | 0 | 0 | 0 | 0 |
| Amikacin | 3 (43) | 18 (69) | 71 (83) | 6 (75) | 1 (50) | 0 | 99 (76) |
| Aztreonam | 1 (14) | 1 (4) | 1 (1) | 3 (38) | 0 | 0 | 6 (5) |
| Ceftazidime | 0 | 0 | 2 (2) | 0 | 0 | 1 (50) | 3 (2) |
| Cefotaxime | 1 (14) | 0 | 0 | 0 | 0 | 0 | 1 (1) |
| Ciprofloxacin | 0 | 6 (23) | 3 (4) | 8 (100) | 0 | 1 (50) | 18 (14) |
| Cefuroxime | 0 | 0 | 0 | 0 | 0 | 0 | 0 |
| Cefazolin | 0 | 0 | 0 | 0 | 0 | 0 | 0 |
| Cefepime | 0 | 0 | 4 (5) | 3 (38) | 1 (50) | 0 | 8 (6) |
| Cefoxitin | 0 | 0 | 1 (1) | 0 | 0 | 0 | 1 (1) |
| Gentamicin | 2 (29) | 13 (50) | 33 (38) | 1 (13) | 1 (50) | 1 (50) | 51 (39) |
| Levofloxacin | 0 | 6 (23) | 0 | 4 (50) | 1 (50) | 1 (50) | 12 (9) |
| Tobramycin | 0 | 3 (12) | 1 (1) | 0 | 0 | 0 | 4 (3) |
| Piperacillin/tazobactam | 0 | 0 | 0 | 0 | 0 | 1 (50) | 1 (1) |
| TMP/SMX | 0 | 3 (12) | 24 (28) | 2 (25) | 1 (50) | 0 | 30 (23) |
| Tigecycline | 5 (71) | 13 (50) | 32 (37) | 2 (25) | 1 (50) | 1 (50) | 54 (41) |
| Tetracycline | 1 (14) | 2 (8) | 6 (7) | 3 (38) | 0 | 0 | 12 (9) |
| Ticarcillin/clavulanate | 0 | 1 (4) | 0 | 0 | 0 | 0 | 1 (1) |

**Abbreviations**: OXA - Oxacillin carbapenemases, NDM - New Delhi metallo-beta-lactamase enzyme**,** KPC - Klebsiella pneumoniae Carbapenemase, VIM - Verona integron-encoded metallo-beta-lactamase, IMP - Imipenemase metallo-beta-lactamase, GES - Guiana extended spectrum, TMP/SMX – Trimethoprim/sulfamethoxazole.

Additional file 1: Table S2. Comparison of antibiotics susceptibility test between KPC and NDM

| Susceptibility | NDM  (N=26) | KPC  (N=86) | P-value |
| --- | --- | --- | --- |
| Ampicillin | 0 | 1 (1) | >0.99 |
| Ampicillin/sulbactam | 0 | 0 | - |
| Amikacin | 18 (69) | 71 (83) | 0.34 |
| Aztreonam | 1 (4) | 1 (1) | 0.41 |
| Ceftazidime | 0 | 2 (2) | >0.99 |
| Cefotaxime | 0 | 0 | - |
| Ciprofloxacin | 6 (23) | 3 (4) | 0.02 |
| Cefuroxime | 0 | 0 | - |
| Cefazolin | 0 | 0 | - |
| Cefepime | 0 | 4 (5) | 0.20 |
| Cefoxitin | 0 | 1 (1) | >0.99 |
| Gentamicin | 13 (50) | 33 (38) | 0.01 |
| Levofloxacin | 6 (23) | 0 | <0.001 |
| Tobramycin | 3 (12) | 1 (1) | 0.91 |
| Piperacillin/tazobactam | 0 | 0 | - |
| TMP/SMX | 3 (12) | 24 (28) | 0.09 |
| Tigecycline | 13 (50) | 32 (37) | 0.64 |
| Tetracycline | 2 (8) | 6 (7) | 0.82 |
| Ticarcillin/clavulanate | 1 (4) | 0 | 0.23 |

**Abbreviations**: NDM – New Delhi metallo-beta-lactamase enzyme, KPC - Klebsiella pneumoniae Carbapenemase, TMP/SMX – Trimethoprim/sulfamethoxazole.

Additional file 1: Table S3. Antibiotics regimen for treating carbapenemase-producing Enterobacteriaceae bloodstream infection

| **Characteristics** | **Total**  **(N=131)** | **Survivor**  **(N=69)** | **Non-survivor**  **(N=62)** | **P** |
| --- | --- | --- | --- | --- |
| Combination therapy |  |  |  | 0.68 |
| - Monotherapy | 32 (30) | 18 (29) | 14 (30) | 0.88 |
| - Double combination therapy | 67 (62) | 40 (65) | 27 (59) | 0.54 |
| - Triple combination therapy | 9 (8) | 4 (7) | 5 (11) | 0.49 |
| Monotherapy |  |  |  |  |
| Amikacin | 6 (6) | 4 (7) | 2 (4) | 0.96 |
| Colistin | 12 (11) | 5 (8) | 7 (15) | 0.39 |
| Gentamicin | 1 (1) | 0 | 1 (2) | 0.88 |
| Tigecycline | 1 (1) | 0 | 1 (2) | 0.88 |
| Others* | 12 (11) | 9 (15) | 3 (7) | 0.32 |
| Double combination therapy |  |  |  |  |
| Amikacin + colistin | 8 (7) | 3 (5) | 5 (11) | 0.42 |
| Amikacin + carbapenem | 7 (7) | 3 (5) | 4 (9) | 0.68 |
| Colistin + gentamicin | 4 (4) | 3 (5) | 1 (2) | 0.83 |
| Colistin + carbapenem | 14 (13) | 7 (11) | 7 (15) | 0.76 |
| Colistin + tigecycline | 4 (4) | 2 (3) | 2 (4) | 1.00 |
| Carbapenem + gentamicin | 1 (1) | 1 (2) | 0 | 1.00 |
| Tigecycline + meropenem | 1 (1) | 1 (2) | 0 | 1.00 |
| Other double combination** | 28 (26) | 20 (32) | 8 (17) | 0.13 |
| Triple combination therapy |  |  |  |  |
| Amikacin + colistin + meropenem | 2 (2) | 0 | 2 (4) | 0.35 |
| Colistin + meropenem + gentamicin | 2 (2) | 0 | 2 (4) | 0.35 |
| Colistin + meropenem + tigecycline | 2 (2) | 2 (3) | 0 | 0.61 |
| Other triple combination*** | 3 (3) | 2 (3) | 1 (2) | 1.00 |
| Amikacin-based therapy | 33 (31) | 17 (27) | 16 (38) | 0.53 |
| Colistin-based therapy | 58 (54) | 28 (45) | 30 (65) | 0.05 |

Note: Data presented are numbers (%) of patients, unless otherwise indicated

*** Others:** meropenem, quinolone, TMP/SMX

**** Other double combination:** amikacin + aztreonam, amikacin + cefepime, amikacin + quinolone, colistin + ampicillin/sulbactam, colistin + cefepime, colistin + piperacillin/tazobactam, colistin + quinolone, colistin + TMP/SMX, carbapenem + fosfomycin, carbapenem + aztreonam, carbapenem + TMP/SMX, carbapenem + quinolone, quinolone + aztreonam, quinolone + piperacillin/tazobactam, tigecycline + quinolone, tigecycline + TMP/SMX

***** Other triple combination:** amikacin + carbapenem +TMP/SMX, carbapenem + ampicillin/sulbactam + fosfomycin
